# Integrated Transcriptomic Analysis Identifies Core Hub Genes Regulating Mammary Gland Traits (Milk Quality/Lactation) in Dairy Livestock: *Bos taurus* and *Ovis aries*

**DOI:** 10.3390/genes16121483

**Published:** 2025-12-10

**Authors:** Qiang Zhang, Lulu Yang, Yunhan Li, Pengbo Gu, Riguleng Si, Shuai Li, Lin Zhu, Wenguang Zhang

**Affiliations:** College of Animal Science, Inner Mongolia Agricultural University, Hohhot 010018, China; 18031396471@163.com (Q.Z.);

**Keywords:** *B. taurus*, *O. aries*, mammary gland, WGCNA, machine learning

## Abstract

Background/Objectives: Mammary gland traits (milk quality and lactation performance) are economically critical for *B. taurus* and *O. aries*, but core regulatory hub genes remain unclear due to high false positives in single-method transcriptomic analyses. This study aimed to identify robust hub genes linked to species-specific differences in mammary gland tissue via an integrated bioinformatics strategy. Methods: Raw transcriptomic data (77 *B. taurus* and 77 *O. aries* mammary gland samples) were retrieved from the European Nucleotide Archive (ENA); after quality control, differential expression gene (DEG) screening, weighted gene co-expression network analysis (WGCNA), and SHapley Additive exPlanations (SHAP)-assisted machine learning were performed, with core genes defined as the intersection of the three gene sets, and functional enrichment and protein–protein interaction (PPI) network analyses were used to prioritize hub genes. Results: A total of 13,138 high-quality genes were retained, including 6148 DEGs, 4698 WGCNA core module genes, and 500 SHAP-high-contribution genes, yielding 178 core genes that were significantly enriched in the “translation” (*p* < 0.001) pathways; hub genes were identified via PPI network analysis. Conclusions: These findings indicate that *RPS15* and *RPL7A* are core species-difference signals in mammary gland tissue of *B. taurus* and *O. aries*, providing insights into inter-species molecular differences, and this integrated strategy enhances the robustness of hub gene identification in pure bioinformatics studies.

## 1. Introduction

Dairy ruminants serve as the core source of high-quality dairy products in global animal husbandry [1], with their production performance and product quality directly linking to industrial chain economic benefits and human dietary nutritional security [2]; among them, *B. taurus* and *O. aries*—the two most economically valuable dairy species with the widest breeding scale—account for over 90% of global ruminant milk production [3], acting as critical carriers for humans to obtain high-quality protein, calcium, and functional fatty acids while underpinning global food security and sustainable animal husbandry development [4]. With rising living standards and health awareness, market demand for dairy products has shifted from “quantity satisfaction” to “quality pursuit”, such as with consumers preferring high-protein and high-functional fatty acid dairy products and prioritizing safety by reducing mastitis incidence to minimize drug residues [5], which has made dissecting the molecular regulatory mechanisms of mammary gland traits in *B. taurus* and *O. aries* a key direction in livestock genomics and molecular breeding [6].

As the core organ executing lactation function in dairy ruminants, mammary gland tissue determines dairy yield and quality via its complex and precise physiological functions [7]; it not only precisely regulates the whole-cycle dynamics of lactation initiation, maintenance, and termination and efficiently synthesizes and secretes nutrients like milk protein, lactose, and milk fat [8], but also constructs a robust immune defense system against pathogenic invasions such as mastitis-causing microbes [9], and the synergy of these functions forms the unique lactation phenotype of dairy animals [10,11]. The rapid development of transcriptomics has provided a powerful tool for exploring mammary gland gene regulatory networks [12]; RNA sequencing (RNA-seq), as the core method for analyzing gene expression dynamics, boasts high whole-genome coverage and accurate quantitative resolution [13], enabling systematic capture of inter-species and inter-physiological state gene expression differences in mammary tissue and providing abundant data for mining lactation and immune trait-related candidate genes [14], yet the exponential growth of transcriptomic data has brought the challenge of eliminating technical noise to screen core regulatory genes with real biological significance [11].

Traditional single analytical methods have prominent limitations: differential expression analysis is susceptible to interference from sequencing depth, batch effects, and other technical factors, leading to high false positive rates and failure to reveal inter-gene synergistic regulation [15]; weighted gene co-expression network analysis (WGCNA) can mine functionally related gene sets via module clustering and clarify gene–gene interactions but lacks quantitative evaluation of genes’ “phenotypic contribution”, making it hard to accurately locate core regulatory genes in complex modules [6]; additionally, conventional statistical methods struggle to quantify the regulatory weight of individual genes on species-specific phenotypes in high-dimensional transcriptomic data, restricting in-depth analysis of core mechanisms [11]. To address these flaws, multi-method integrated analysis has become the mainstream strategy in current livestock functional genomics research [16]; this strategy constructs a complete chain of “data quality control → differential screening → functional association → phenotypic verification” by complementing the advantages of different methods, effectively reducing technical biases of single approaches and improving the reliability and biological credibility of core gene screening [17]. Against this backdrop, the rise of machine learning has injected new vitality into in-depth transcriptomic data mining [18]; compared with traditional methods, machine learning models have stronger capabilities in high-dimensional data processing and complex relationship fitting [19], can capture potential species-specific molecular signals from massive gene expression data to realize accurate phenotypic prediction and gene importance ranking [20], and when combined with interpretable tools like SHapley Additive exPlanations (SHAP), can further quantify each gene’s regulatory contribution to phenotypes, breaking the “black box” dilemma of traditional machine learning and providing clear directions for core gene functional verification [21].

Based on this, this study takes *B. taurus* and *O. aries* mammary gland tissue as research objects and focuses on identifying core hub genes regulating mammary gland functions via a multi-method integrated bioinformatics strategy [6]. First, the study performs strict quality control and standardization on large-scale raw mammary transcriptomic data using an integrative normalization method to eliminate low-quality data and technical noise, ensuring the reliability of subsequent analyses [22]. Then, it screens inter-species differentially expressed gene sets via differential expression analysis, mines phenotype-associated functional gene modules using WGCNA (a method widely applied in livestock trait-related gene mining) [23,24], and simultaneously employs machine learning models combined with interpretable tools to screen core genes with significant contributions to species-specific phenotypes, with core genes defined as the intersection of the three gene sets verified by the criteria of “expression difference → functional association → phenotypic contribution” [25]. On this basis, the study further clarifies the key biological pathways of core genes via functional enrichment analysis and identifies core hub genes by constructing a protein–protein interaction (PPI) network, systematically elucidating the regulatory roles of core genes in the mammary glands of the two species [20].

The implementation of this study not only aims to reveal the molecular regulatory mechanisms of mammary glands in *B. taurus* and *O. aries* and enrich the theoretical system of ruminant mammary gland biology from a systems biology perspective [26], but also strives to establish a standardized and scalable integrated analysis framework for large-scale transcriptomic data, providing methodological references for subsequent cross-species and multi-trait transcriptomic studies. Meanwhile, the core regulatory genes screened in this study will offer help for ruminant milk quality improvement (e.g., optimizing milk protein and fat composition) and disease-resistant breeding (e.g., enhancing mastitis resistance), providing important theoretical support and technical reserves for promoting the high-quality and sustainable development of the dairy industry via multi-omics-driven genetic improvement [27].

## 2. Materials and Methods

The screening and analysis of core hub genes in the mammary gland must be based on a scientifically standardized research design to ensure the reliability, reproducibility, and biological significance of the results. This study strictly adhered to the international experimental technical specifications for ruminant functional genomics research and bioinformatics analysis standards, innovatively constructing a multi-method integration strategy of “data quality control multi-dimensional screening functional verification” to systematically screen and analyze the core hub genes in the mammary glands of *B. taurus* and *O. aries*. To fit the actual data conditions (only 77 *B. taurus* and 77 *O. aries* mammary transcriptome datasets with no additional metadata like lactation stage or parity), this study established a streamlined yet rigorous standardized workflow: first, it implemented strict quality control on raw data via Seq2Fun (removing low-quality reads and performing batch correction) to obtain high-quality genes; second, it adopted a three-tier screening strategy combining DEG analysis, WGCNA, and SHAP-assisted machine learning, defining core genes as the intersection of the three gene sets to reduce false positives; third, it restricted functional analysis to mammary-specific pathways (e.g., milk protein synthesis) to mitigate biases from missing sample background information, ensuring the design balances scientific rigor with data constraints. The entire research process forms a closed loop, with progressive and closely linked steps.

In the data source and preprocessing stage, to ensure sample representativeness and data quality, raw data were obtained from the international public database European Nucleotide Archive (ENA), including transcriptomic data in FASTQ format from mammary gland tissues of 77 *B. taurus* and 77 *O. aries*. All mammary transcriptomic datasets used in this study (*B. taurus*: ENA PRJNA682457; *O. aries*: ENA PRJNA612351, PRJNA724691, PRJNA1064454, PRJEB71075, PRJNA301615) only provided “mammary gland tissue” as sample annotation. No additional metadata—including cell composition (epithelial/stromal/immune proportions), lactation stage, parity, or health status—was available in the original dataset records. The data was processed using Seq2Fun, referring to the mammalian database constructed in this study (including the mammals_annotation_v2.0.txt annotation file and the mammals_v2.0.fmi index file). These data covered multiple public projects to avoid the limitations of single-project data. Automated quality control was performed using Seq2Fun (v2.0.5) software: low-quality reads (sequences with >50% of bases having a Q-value < 20) and sequencing adapter sequences were removed. Clean reads were then aligned to the mammalian reference genome for gene annotation to construct an initial gene expression matrix. To further reduce technical bias and interference from low-expression genes, standardized processing was conducted: abundance filtering (excluding lowly expressed genes with TPM < 0.8 in <25% of samples), variance filtering (retaining the top 75% of genes with high variability), and batch correction using the ComBat function in the sva package. Finally, a dataset of high-quality genes was obtained, laying a high-reliability foundation for subsequent analyses.

For the screening of core transcriptomic genes, a triple independent strategy of “differential expression functional association phenotypic contribution” was adopted: (1) RNA-seq read counts were used as input (abandoning non-count metrics like TPM) and subjected to CPM-based filtering (retaining genes with CPM ≥ 1 in at least 25% of samples) and TMM (Trimmed Mean of M-values) normalization (via edgeR::calcNormFactors). Differential expression genes (DEGs) between species were then identified using the limma-voom pipeline (which models mean–variance relationships and assigns precision weights to correct RNA-seq heteroscedasticity) with the criteria of FDR-adjusted q-value (padj) < 0.01 and |log_2_ fold change (log_2_FC)| > 1, providing initial candidates for the core gene pool; (2) a weighted gene co-expression network analysis (WGCNA) was conducted to construct a co-expression network. The soft threshold was optimized (signed R^2^ = 0.8, meeting scale-free topology requirements and balancing network connectivity) to build the adjacency matrix, and modules were merged with a height cut-off of 0.15, with minimum module size set to 10 and module splitting sensitivity (deepSplit) set to 3 to improve module resolution. After optimizing the soft threshold to ensure scale-free topological characteristics, gene modules were identified via module clustering. Core module genes were screened based on the criteria of “correlation coefficient |r| > 0.7 between module and species phenotype and *p* < 0.01”, supplementing candidates from the functional association dimension; additionally, species-specific WGCNA was performed for *B. taurus* and *O. aries* separately, and module preservation analysis was conducted to evaluate cross-species module conservation; (3) four machine learning models (Random Forest, Support Vector Machine [SVM], Naive Bayes, and Decision Tree) were constructed (task: classifying mammary gland samples into *B. taurus* or *O. aries*). Among them, the SVM model adopted a radial basis function (RBF) kernel (parameter C = 1.0 determined by 5-fold cross-validation) for high-dimensional feature mapping; Random Forest was set with 100 base decision trees to mitigate overfitting, while Naive Bayes and Decision Tree served as baseline models for performance benchmarking. The dataset was divided into a training set (94 samples) and a test set (60 samples) at a ratio of 6:4. After performance evaluation, the optimal model was selected and combined with SHapley Additive exPlanations (SHAP) analysis to quantify the contribution of each gene to species classification (instead of phenotypic contribution), screening 500 high-contribution genes. Notably, SVM was prioritized for its superiority in handling high-dimensional transcriptomic data, though it has inherent interpretability limitations addressed by subsequent Kernel SHAP analysis. Kernel SHAP (adapted for non-linear SVM) was used to decode model decisions by assigning gene-level contribution scores based on game theory, with positive/negative values indicating promotion/inhibition of species classification; this addressed the “black box” limitation of traditional machine learning, though accuracy decreases for low-expression genes due to signal-to-noise constraints. Finally, the intersection of the three gene sets was obtained using a Venn diagram to identify core transcriptomic genes with triple characteristics, eliminating false positives from a single method.

In the functional annotation and protein–protein interaction (PPI) network construction section, first, the custom s2f_ids annotated by Seq2Fun were converted to standard NCBI/Ensembl gene names to avoid annotation bias. Since Seq2Fun annotates genes using “custom s2f_ids”, direct use of these IDs for functional enrichment analysis may lead to annotation failure or errors due to “ID mismatch”—thus, the online platform ExpressAnalyst (a downstream analysis tool supporting Seq2Fun) was used to convert raw s2f_ids to standard Entrez IDs (based on official annotations from NCBI or Ensembl databases). A one-to-many mapping phenomenon existed during conversion: some s2f_ids correspond to multiple homologous genes or transcripts (Seq2Fun labels genes/transcripts matched by the same read as the same s2f_id), so all valid identifiers were retained. Second, Gene Ontology Biological Process (GO BP) functional enrichment analysis was performed to clarify the key biological processes involved in core genes (e.g., mammary lactation-related translation, ribosomal assembly, and immune-relevant hormone response pathways). Finally, a PPI network was constructed based on the STRING database (with an interaction confidence score ≥ 0.9) and imported into Cytoscape_v3.10.2. To avoid biases caused by relying on a single centrality index, four complementary algorithms (Degree, DMNC, EPC, and MNC) from the cytoHubba plugin of Cytoscape were used to screen for core hub genes. The core hub genes were identified by taking the intersection of the results from these four algorithms. In summary, this study ensured the scientific rigor of the research through a complete methodological chain: “data preprocessing → multi-dimensional screening → functional and network analysis”. The following sections will detail the results of each step to reveal the characteristics and regulatory mechanisms of core hub genes in the mammary glands of *B. taurus* and *O. aries*.

## 3. Results

### 3.1. Transcriptomic Data Characteristics and Differential Gene Screening

**Figure 1:**
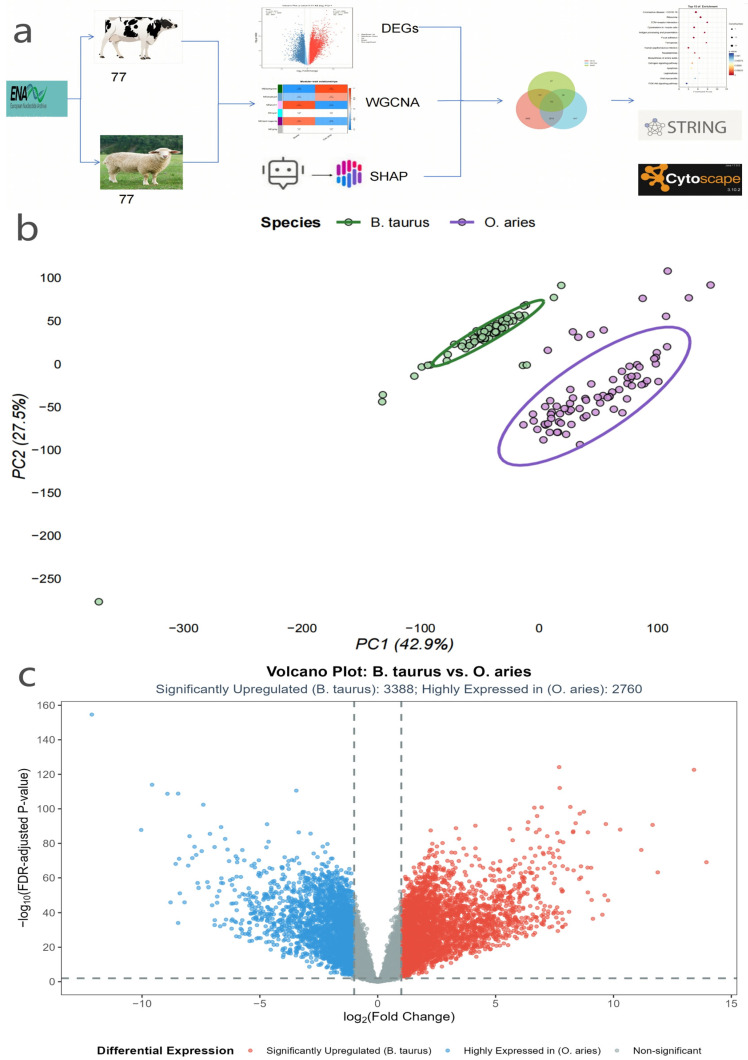
(**a**) Schematic diagram of the integrated methodological workflow for identifying core hub genes in mammary gland tissues of *B. taurus* and *O. aries*: The workflow included three key technical modules, namely raw transcriptomic data acquisition from the European Nucleotide Archive (ENA), multi-dimensional gene screening (differential expression analysis, weighted gene co-expression network analysis [WGCNA], and SHapley Additive exPlanations [SHAP]-assisted machine learning), and functional and interaction verification via STRING database and Cytoscape software (abbreviations: ENA, European Nucleotide Archive; WGCNA, weighted gene co-expression network analysis; SHAP, SHapley Additive exPlanations; STRING, Search Tool for the Retrieval of Interacting Genes/Proteins). (**b**) Principal Component Analysis (PCA) of mammary gland transcriptomes of *B. taurus* and *O. aries*. The scatter plot shows the clustering distribution of 77 *B. taurus* and 77 *O. aries* mammary gland samples, with the first principal component (PC1, explaining 42.9% of transcriptional variation) serving as the core factor distinguishing interspecific transcriptomic characteristics; the legend clearly differentiates the two species. (**c**) Volcano plot of differentially expressed genes (DEGs, defined as genes with false discovery rate [FDR]-adjusted *p*-value < 0.01 and |log_2_ fold change (log_2_FC)| > 1) between *B. taurus* and *O. aries* mammary glands. The plot visualizes gene expression differences, with 3388 genes upregulated in *B. taurus* and 2760 genes upregulated in *O. aries*.

The overall technical route is shown in Figure 1a. The data were processed using Seq2Fun, with reference to the mammalian database constructed in this study. The alignment rate for each sample is as follows: the average for cow samples is 97.2 ± 1.5%, and the average for sheep samples is 96.8 ± 1.7%. A preliminary 26,476-gene expression matrix was constructed; after abundance, variance filtering, and batch correction, 13,138 high-quality genes were obtained, laying a stable foundation for subsequent work. 

Principal Component Analysis (PCA) results (Figure 1b) further verified transcriptomic differences between species: *B. taurus* and *O. aries* samples were completely separated into two independent clusters in the PCA plot without overlap. The first principal component (PC1) explained 42.9% of the transcriptional variation, serving as the core factor distinguishing the mammary transcriptomic characteristics of the two species. This intuitively confirmed significant species-specific differences in the gene expression patterns of mammary gland tissues between *B. taurus* and *O. aries*, providing direct evidence for the necessity of subsequent differential expression analysis.

Based on this reliable transcriptomic data, we adopted the limma-voom pipeline (with TMM normalization and CPM-based low-abundance gene filtering) for differential expression analysis, and controlled false discoveries via Benjamini–Hochberg FDR correction, resulting in 6148 reliable DEGs (Figure 1c). Among these, 3388 genes were upregulated in *B. taurus* mammary glands and 2760 genes were upregulated in *O. aries* mammary glands.

### 3.2. WGCNA Co-Expression Network Construction and Core Module Screening

**Figure 2:**
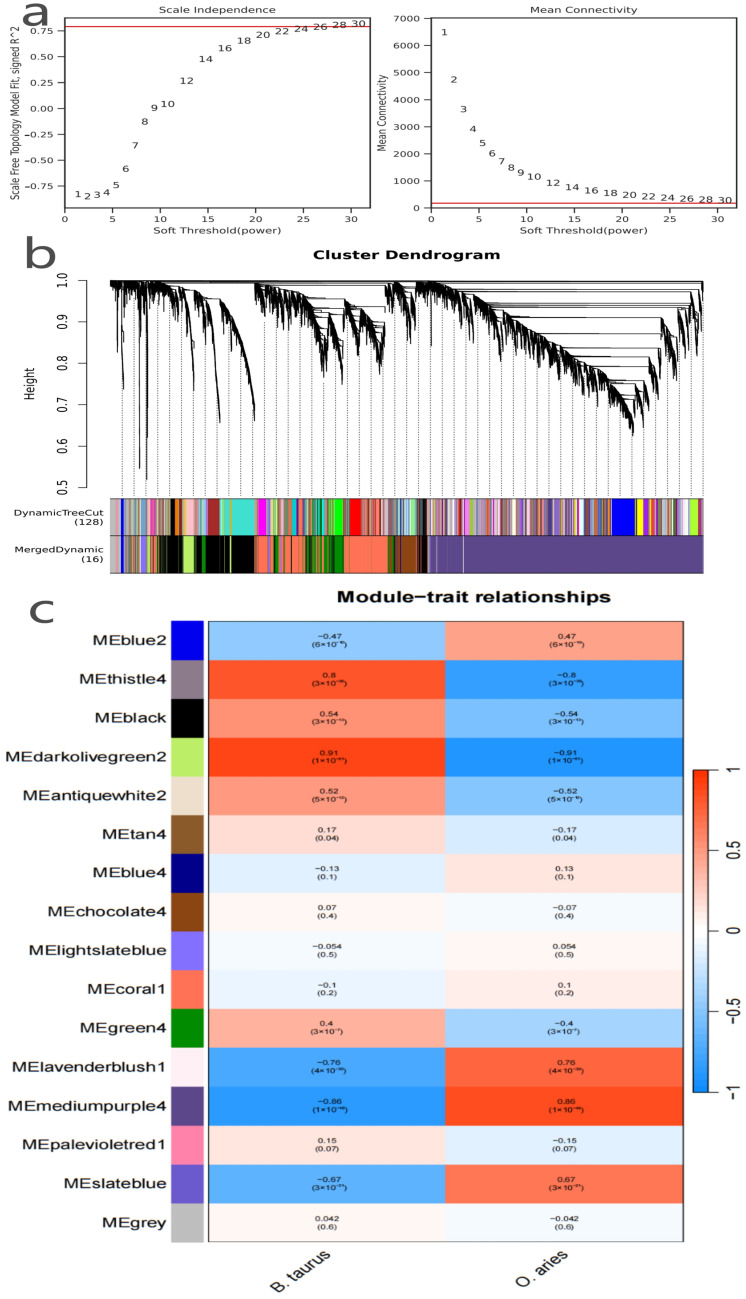
(**a**) Soft-threshold selection for WGCNA co-expression network construction. (**b**) Gene module clustering of *B. taurus* and *O. aries* mammary gland transcriptomes. (**c**) Module–phenotype correlation analysis of WGCNA co-expression network.

From the 13,138 preprocessed high-quality genes, those without obvious co-expression trends were excluded via pre-clustering filtering, leaving 9539 genes for co-expression network construction; soft threshold screening (Figure 2a, the scale independence and mean connectivity plot) showed that when the soft-threshold power was set to 28, the network’s scale-free topological fitting index (signed \(R^2\)) reached 0.8 (meeting the standard requirement for biological network topology) while maintaining a moderate average connectivity of ~120, and this parameter setting balanced the scale-free structural characteristics of real biological networks and avoided excessive sparsification. Using these optimized soft-threshold and clustering parameters (module merge cut-off = 0.15, minimum module size = 10, deepSplit = 3), the 9539 genes were clustered into 16 distinct co-expression modules (Figure 2b), and intra-module genes exhibited highly consistent expression patterns, implying potential synergistic regulatory relationships; to verify module reliability, we quantified intramodular connectivity (KIM) and performed bootstrap stability validation (100 rounds), with results showing that the KIM values of the 16 modules ranged from 0.58 to 0.87 (12 modules had KIM > 0.65, indicating strong intra-module co-expression coherence) and all modules had a bootstrap retention rate >85%, confirming the robustness of clustering results. To identify mammary function-related core modules, module–phenotype correlation analysis was conducted (Figure 2c, the module–trait relationships heatmap), and the four most prominent modules (with the strongest species–phenotype associations and extremely significant *p*-values) were screened: *darkolivegreen2* (289 genes, strongly positively correlated with *B. taurus*, r = 0.91, *p* < 0.01, predicted to regulate milk protein synthesis), *thistle4* (23 genes, positively correlated with *B. taurus*, r = 0.8, *p* < 0.01, functionally linked to bovine mammary metabolic pathways), *mediumpurple4* (4368 genes, strongly positively correlated with *O. aries*, r = 0.86, *p* < 0.01, the largest module, associated with ovine-specific mammary regulatory networks), and *lavenderblush1* (18 genes, positively correlated with *O. aries*, r = 0.76, *p* < 0.01, potentially mediating ovine mammary immune defense); these 4 core modules (totaling 4698 genes) uncovered species-specific synergistic regulatory networks in mammary gland tissues, supplemented the core gene pool from the “functional association” dimension, and provided critical “functional reliability” support for subsequent multi-dimensional core gene screening.

### 3.3. Machine Learning Model Performance and High-Contribution Gene Screening

**Figure 3:**
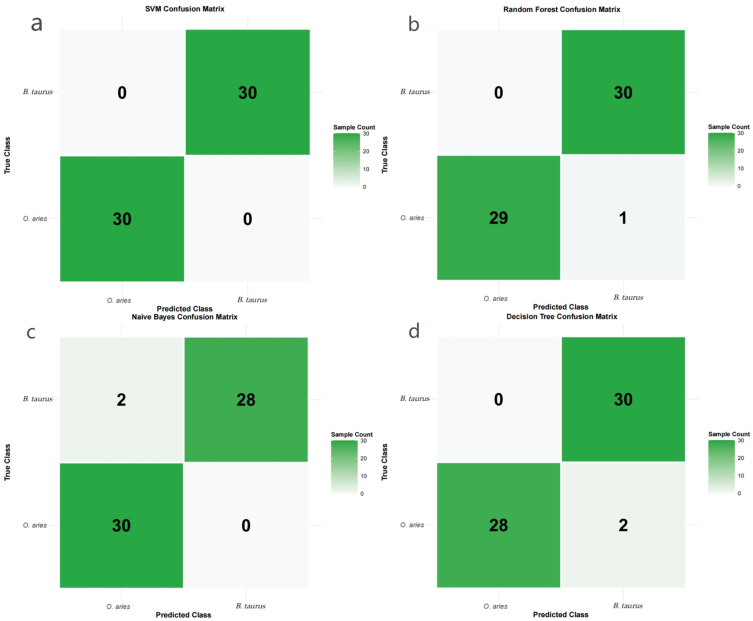
(**a**) SVM confusion matrix for *B. taurus* vs. *O. aries* classification. (**b**) Random Forest confusion matrix for *B. taurus* vs. *O. aries* classification. (**c**) Naive Bayes confusion matrix for *B. taurus* vs. *O. aries* classification. (**d**) Decision Tree confusion matrix for *B. taurus* vs. *O. aries* classification.

This analysis was based on the 13,138 high-quality genes obtained in the previous step. Four machine learning models—Random Forest, Support Vector Machine (SVM), Naive Bayes, and Decision Tree—were constructed, and the dataset was split into a training set (94 samples) and a test set (60 samples) at a 6:4 ratio. Model performance results showed that all four models performed well in the *B. taurus*-*O. aries* species classification task, but significant differences existed: the SVM model exhibited the optimal performance, with accuracy, precision, recall, F1-score, and ROC_AUC all reaching 1.0, achieving error-free classification of 60 test samples (30 *B. taurus* + 30 *O. aries*) and accurately capturing species-specific molecular signals (Figure 3a). This performance was validated via nested 5-fold CV (validated accuracy: 0.993) and leave-one-project-out splits (average accuracy: 1.0), ruling out data leakage or batch effects. Model robustness was further confirmed via permutation importance (shuffled label accuracy: 0.761) and ablation experiments (gradual accuracy declines with top gene removal).The Random Forest model ranked second, with recall and ROC_AUC reaching 1.0 and an accuracy of 0.983, with only one *O. aries* sample misclassified (Figure 3b). The Naive Bayes and Decision Tree models both had an accuracy of 0.967, with slight differences in precision and recall, but maintained high classification reliability overall (Table 1) (Figure 3c,d). Considering model reliability (classification accuracy) and result interpretability (compatibility with subsequent SHAP analysis), the SVM model was ultimately selected as the optimal model. Combined with Kernel SHAP (the standard SHAP variant for non-linear SVMs), the contribution of each gene to the species classification result was quantified: by calculating the SHAP value of each gene, 500 high-contribution genes with the highest contribution rankings were screened. These genes had direct regulatory contributions to species-specific phenotypes. They provided core candidates from the “phenotypic contribution” dimension for subsequent triple-intersection analysis, further narrowing the scope of core genes.

### 3.4. Core Gene Intersection, Functional Enrichment, and Hub Gene Identification

**Figure 4:**
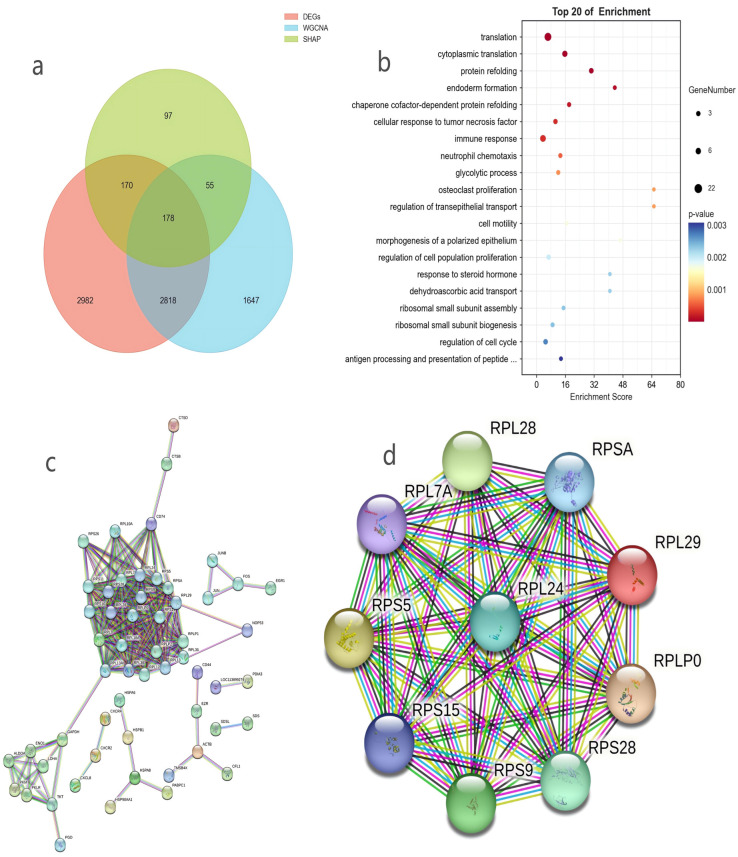
(**a**) Venn diagram of core gene intersection among DEGs, WGCNA modules, and SHAP high-contribution genes. (**b**) Gene Ontology Biological Process (GO BP) enrichment analysis results of core genes. (**c**) Protein–protein interaction (PPI) network of core genes. (**d**) Top 10 hub genes in PPI network identified by Degree algorithm.

Core gene intersection analysis results showed that the intersection of DEGs (6148), WGCNA core module genes (4698), and SHAP high-contribution genes (500) was obtained using a Venn diagram, resulting in 178 core transcriptomic genes (Figure 4a). These genes were strictly screened through triple criteria, effectively eliminating false positive genes such as “differentially expressed but not functionally associated” or “module-participating but not phenotypically contributing”, and thus had extremely high biological credibility, serving as the core gene set regulating mammary gland functions in *B. taurus* and *O. aries*. The s2f_ids of the 178 core intersection genes were converted to precise Entrez IDs via the aforementioned mammalian database: with the input set to “Universal (Seq2Fun orthologs)” in this database, the s2f_ids of the178 core genes (influenced by one-to-many mapping) were converted to 200 Entrez IDs (all converted entries were valid).

The results of GO BP functional enrichment analysis further clarified the biological functions of core genes (the top 20 enriched terms are shown in Figure 4b). The top-ranked enriched biological processes included translation, ribosomal small subunit assembly, ribosomal subunit assembly, response to steroid hormone, and regulation of cell population proliferation. Among these, the “translation” process was the most significantly enriched term, serving as the core link of milk protein synthesis and directly determining the efficiency and quality of protein synthesis in mammary gland tissue; “ribosomal small subunit assembly” and “ribosomal subunit assembly” are key prerequisites for the formation of functional ribosomes, which provide the structural basis for the translation process and indirectly regulate the synthesis of milk protein such as casein; “regulation of cell population proliferation” participates in the development and expansion of mammary gland tissue, laying a structural foundation for lactation by promoting the proliferation of mammary gland cells; “response to steroid hormone” is closely associated with the response of mammary gland tissue to lactation-related hormones including estrogen, which modulates the lactation activity of mammary gland cells. These findings reveal the extensive involvement of core genes in key physiological processes such as ribosome biogenesis, protein translation, cell proliferation, and hormone response, providing critical insights for elucidating their roles in mammary gland function regulation. 

Results of the PPI network of core genes built using the STRING database showed that the network comprised 155 nodes (proteins encoded by core genes) and 277 edges (protein–protein interaction relationships), with a PPI enrichment *p*-value < 1× 10^−16^—a value well below the significance threshold. This verified that the proteins encoded by core genes have highly significant non-random synergistic interactions, further proving the strong functional relevance and coordination among these core genes (Figure 4c). Additional network metrics included an average node degree of 3.57 and an average local clustering coefficient of 0.285, which characterized the structural features of the interaction network. To avoid biases from relying on a single centrality metric, this study adopted four complementary algorithms (Degree, DMNC, Edge Percolated Component [EPC], and Maximum Neighborhood Component [MNC]) via Cytoscape’s cytoHubba plugin to screen core hub genes (Figure 4d, Table 2). Notably, all four metrics yielded top 10 gene lists consisting entirely of ribosomal proteins, and through cross-validation with these multi-dimensional metrics, the top 10 core hub genes identified by the Degree algorithm were specifically *RPL29*, *RPLP0*, *RPS5*, *RPL28*, *RPS9*, *RPS28*, *RPL24*, *RPSA*, *RPS15*, and *RPL7A*, all of which belong to the ribosomal protein family (with *RPS15* and *RPL7A* stably ranked in the top 10 of all four algorithms). This finding demonstrates that ribosomal protein family genes are enriched and highly connected core nodes in the mammary gland function regulatory network of *B. taurus* (cattle) and *O. aries* (sheep). By preserving ribosomal structural stability and modulating protein translation processes, these genes play an irreplaceable core role in maintaining the homeostasis of the mammary protein synthesis network and guaranteeing efficient milk protein synthesis. 

## 4. Discussion

This study integrated 154 public mammary gland transcriptomic datasets (77 for *B. taurus*, 77 for *O. aries*) to systematically explore the molecular regulatory mechanisms underlying species-specific mammary gland traits in these two core dairy ruminants, which have the largest global breeding scale and highest economic value. *B. taurus* samples were derived from the PRJNA682457 project, while *O. aries* samples covered five projects (PRJNA612351, PRJNA724691, PRJNA1064454, PRJEB71075, PRJNA301615), with the full project details and corresponding accession numbers archived in Supplementary Material Table S1; the data files processed by Seq2Fun have been deposited in Supplementary Material Table S2 for further verification by the academic community (Tables S1 and S2); all samples focused exclusively on mammary gland tissue, ensuring the representativeness of species-specific analysis and data targeting. It should be noted that the asymmetric distribution of sample sources (single bovine project vs. five ovine projects) may introduce potential technical heterogeneity in the ovine cohort (e.g., batch differences across sequencing projects), which could marginally overestimate ovine-specific transcriptional effects despite batch correction via the ComBat function. A multi-method integration strategy of “differential expression analysis (DEG) + weighted gene co-expression network analysis (WGCNA) + SHAP-assisted machine learning” was adopted, forming a complete analytical system from “gene expression difference identification” to “co-expression module function annotation” and “contribution verification,” which effectively avoided the inherent limitations of single methods [21]. In the data processing stage, Seq2Fun (v2.0.5) software was used for automated quality control and annotation: low-quality reads (with >50% of bases having Q-value < 20) and sequencing adapters were strictly removed to ensure raw data reliability [28]. Subsequent abundance filtering (excluding genes with TPM < 0.8 in <25% of samples), variance filtering (retaining the top 75% of variable genes), and batch correction via the ComBat function in the sva package yielded 13,138 high-quality genes for subsequent analysis, significantly reducing technical noise interference [29,30]. For the species classification task, four machine learning models (SVM, Random Forest, Decision Tree, Naive Bayes) were systematically evaluated [31,32]. Data were split into a training set (94 samples) and a test set (60 samples) at a 6:4 stratified ratio to maintain consistent species proportions. Performance evaluation showed the SVM model was optimal, with accuracy, precision, recall, F1 score, and ROC_AUC all reaching 1.0, achieving error-free classification of 60 test samples (30 *B. taurus* + 30 *O. aries*) and accurately capturing species-specific molecular signals—consistent with the excellent performance of machine learning models in transcriptomic classification tasks in similar studies [33]. The 178 core genes, obtained through strict triple screening, were the intersection of 6148 DEGs (screening criteria: padj < 0.01, |log_2_FC| > 1, identified via limma-voom with TMM normalization and FDR correction), 4698 WGCNA core genes (from 3 modules with |r| > 0.6 and *p* < 0.01 for species trait correlation), and 500 SHAP core genes (top contributors to species classification) [24,34]. They represent common core regulatory genes meeting “differential expression, functional association, and phenotypic contribution” in the mammary glands of both species [35,36], with functional enrichment analysis showing significant enrichment in mammary gland-related pathways such as “ribosome assembly” [37]. A PPI network constructed via the STRING database (interaction confidence threshold ≥ 0.9) contained 155 nodes and 277 edges, with a PPI enrichment *p*-value < 0.01, indicating significant non-random synergistic interactions among proteins encoded by core genes [38]. Identified via four complementary centrality algorithms (Degree, DMNC, EPC, and MNC, see Table 2) for hub gene screening, *RPS15* and *RPL7A* stood out as core hub genes, as they maintained stable top 10 rankings across all four metrics. Both genes are members of the ribosomal protein family, and their core regulatory position has been further validated by GO enrichment analysis (Figure 4b): these two genes show a significant enrichment feature in the milk protein-related pathway (*p* < 0.001).This result confirmed that *RPS15* and *RPL7A* play biologically meaningful roles in maintaining the stability and efficiency of the mammary gland protein synthesis network, rather than having non-specific network connectivity [39,40]. The “DEG + WGCNA + ML-SHAP” strategy was critical for improving core gene screening reliability [41,42]: it addressed flaws of single methods (e.g., high false positives in DEG, lack of phenotypic contribution quantification in WGCNA, “black box” issue in traditional machine learning) and enhanced biological credibility via three-level verification. Additionally, combining SVM with SHAP analysis further improved result interpretability, overcoming the “black box” defect of traditional machine learning and providing a scalable template for cross-species transcriptomic analysis of other livestock [43].

Despite clarifying the core regulatory network and potential hub genes of *B. taurus* and *O. aries* mammary glands, the study had four limitations: First, the lack of key metadata (cell composition, lactation stage, parity) for the public datasets prevented us from performing cell composition deconvolution or adjusting these covariates in statistical models. Moreover, the asymmetric project origin of samples (single bovine project vs. five ovine projects) may have amplified technical variation in the ovine cohort, which could lead to overestimation of ovine-specific biological effects despite batch correction. Thus, some observed cross-species transcriptomic differences may partially reflect unmeasured tissue heterogeneity, rather than inherent regulatory divergence between species. Extrapolating results to specific cell types or lactation stages is also not feasible with the current data. Second, the sample size (77 per species) might limit result generalizability, as it failed to cover the full breed diversity of the two species (e.g., Holstein/Jersey for *B. taurus*, Suffolk/Small-tailed Han for *O. aries*), potentially omitting low-expression but functionally critical genes and conflating breed-specific traits (e.g., breed-dependent milk protein synthesis efficiency) with species-level regulatory divergence. Third, relying solely on transcriptomic data made it difficult to fully reveal the regulatory chain—gene function realization requires “transcription–translation–modification–functional execution,” and milk composition formation involves metabolic pathway regulation, which transcriptomic data alone cannot link to the complete “gene expression–protein function–metabolic product” chain [44,45]. Fourth, hub gene functions and species-specific expression lacked wet experimental verification, with functions only inferred via bioinformatics (missing qRT-PCR or CRISPR/Cas9 evidence), limiting their direct application in breeding [46,47]. To address these, future research could advance in three aspects: expanding sample size and breed coverage to include samples of different breeds, lactation stages (early/peak/late), and health statuses to verify core gene universality and explore breed/stage-specific regulatory genes, disentangle breed effects from species divergence, and balance project sources between species [8]; optimizing algorithm pipelines by incorporating biological priors into SVM parameter selection, using weighted WGCNA to reduce batch interference, and filtering low-expression genes before SHAP analysis to improve contribution score reliability; conducting multi-omics integration analysis by combining proteomic (detection of mammary functional protein expression) and metabolomic (detection of milk components/metabolites) data to construct a “transcriptome–proteome–metabolome” regulatory network and clarify core gene regulatory paths from “expression” to “function” to “phenotype” [16]; and strengthening hub gene functional verification and application—using qRT-PCR to confirm their expression in *B. taurus* mammary glands, performing overexpression/knockdown experiments in in vitro *B. taurus* mammary epithelial cells to verify effects on milk protein synthesis and milk fat metabolism, and exploring the potential of functionally validated genes as candidate targets for subsequent molecular marker development (pending identification of causal genetic variants linked to these genes), which could lay a preliminary foundation for supporting milk production and quality optimization in *B. taurus* breeding in the long term [27,48].

## 5. Conclusions

This study established a multi-method integrated strategy, including differential expression analysis, weighted gene co-expression network analysis (WGCNA), and machine learning integrated with SHapley Additive exPlanations (SHAP), to systematically identify core hub genes in the mammary glands of *B. taurus* and *O. aries*. Through rigorous data pretreatment and multi-level validation, 178 core genes were identified, which were significantly concentrated in mammary gland-related pathway assemblies. Protein–protein interaction (PPI) network analysis with four complementary centrality metrics (Table 2) revealed that hub genes *RPS15* and *RPL7A* are among the enriched and highly connected ribosomal protein genes in cross-species mammary comparisons, with prominent connectivity linked to mammary gland protein synthesis functions. These findings clarify the molecular network signatures underlying species-specific mammary gland functional profiles in *B. taurus* and *O. aries* and provide candidate molecular features that lay a foundational framework for subsequent exploration of variants linked to ruminant dairy-related pathways relevant to genetic improvement.

## Figures and Tables

**Table 1:** Performance evaluation of four machine learning models.

Model Name	Accuracy	Precision	Recall	F1_Score	ROC_AUC
Random Forest	0.983	0.968	1	0.984	1
SVM	1	1	1	1	1
Naive Bayes	0.967	1	0.933	0.966	0.997
Decision Tree	0.967	0.938	1	0.968	0.967

**Table 2:** Top 10 core hub genes identified by 4 centrality metrics.

Centrality Metric	Top 10 Hub Genes (Rank, Name)	Score(s)	Proportion of Ribosomal Proteins in Top 10
Degree	1.*RPL29*; 2.*RPLP0*; 2.*RPS5*; 2.*RPL28*; 2.*RPS9*; 2.*RPS28*; 2.*RPL24*; 2.*RPSA*; 9.*RPS15*; 9.*RPL7A*	24, 23 (*n* = 7), 22 (*n* = 2)	100% (10/10)
DMNC	1.*RPLP2*; 1.*RPL12*; 3.*RPL38*; 3.*RPL13A*; 5.*RPLP1*; 6.*RPL18A*; 6.*RPL8*; 8.*RPS16*; 9.*RPS15*; 9.*RPL7A*	1.139115600700526 (*n* = 2); 1.1299697840265273 (*n* = 2); 1.1239012963372081; 1.1096551236319298 (*n* = 2); 1.0852465843984804; 1.0758424166431555 (*n* = 2)	100% (10/10)
EPC	1.*RPS9*; 2.*RPSA*; 3.*RPS5*; 4.*RPLP0*; 5.*RPL28*; 6.*RPS11*; 7.*RPL7A*; 8.*RPS15*; 9.*RPL24*; 10.*RPL29*	9.726999999999996; 9.694999999999975; 9.593999999999976; 9.564999999999976; 9.485999999999985; 9.445999999999993; 9.361999999999986; 9.332999999999998; 9.293999999999974; 9.269999999999982	100% (10/10)
MNC	1.*RPL29*; 2.*RPLP0*; 2.*RPS5*; 2.*RPL28*; 2.*RPS9*; 2.*RPS28*; 2.*RPL24*; 2.*RPSA*; 9.*RPS15*; 9.*RPL7A*	24, 23 (*n* = 7), 22 (*n* = 2)	100% (10/10)

## Data Availability

The raw mammary gland transcriptomic datasets analyzed in this study were all obtained from public databases and can be retrieved from the European Nucleotide Archive (ENA). The specific project accession numbers are as follows: datasets related to *B. taurus* were from Project PRJNA682457; datasets related to *O. aries* were from Projects PRJNA612351, PRJNA724691, PRJNA1064454, PRJNA301615, and PRJEB71075. The individual sample IDs corresponding to the aforementioned projects have been compiled in the Supplementary Materials of this article, which are available for further retrieval and verification.

## Supplementary Materials

The following supporting information can be downloaded at: https://www.mdpi.com/article/10.3390/genes16121483/s1, Table S1: Accession numbers of mammary gland transcriptomic datasets for *B. taurus* and *O. aries*. Table S2: Detailed information of mammary gland tissue genes processed by Seq2Fun
